# Clinical, Histopathological, and Molecular Prognostic Factors Associated With Survival and Disease Progression in Adult Patients With Primary and Secondary Thyroid Lymphomas: Scoping Review Protocol

**DOI:** 10.2196/73164

**Published:** 2026-02-20

**Authors:** Ana M Lemos-Rodriguez, Juan Pablo Lenis-Gonzalez, Oriana Arias-Valderrama, Elizabeth Arrieta, Guillermo Edinson Guzman, Andres Octavio Garcia

**Affiliations:** 1 Departamento Medicina Interna, Unidad de Hemato-oncología, Fundación Valle del Lili, Cra 98 No. 18–49, Santiago de Cali, Colombia, 57 3310909; 2 Centro de Investigaciones Clínicas, Fundación Valle del Lili, Cali, Valle del Cauca Department, Colombia; 3 Facultad de Ciencias de la Salud, Universidad Icesi, Santiago de Cali, Colombia; 4 Departamento Medicina Interna, Unidad de endocrinología, Fundación Valle del Lili, Cali, Valle del Cauca Department, Colombia

**Keywords:** prognostic factors, scoping review methodology, thyroid lymphoma, secondary thyroid lymphoma, primary thyroid lymphoma

## Abstract

**Background:**

To identify the key prognostic factors in patients with primary and secondary thyroid lymphoma, focusing on clinical, histopathological, biological, and genetic components, as these factors remain unclear.The key prognostic factors in patients with primary and secondary thyroid lymphoma remain unclear, particularly regarding clinical, histopathological, biological, and genetic components.

**Objective:**

This study aimed to analyze and synthesize the available evidence to identify factors that may influence the prognosis of patients with primary and secondary thyroid lymphoma.

**Methods:**

This review will include indexed databases such as Scopus, Ovid-MEDLINE, and EMBASE as the primary search sources. The search strategy was applied during the period from June 1, 2024, to September 1, 2024, encompassing all available literature. Eligibility criteria include observational studies of human patients aged 18 years or older, with a diagnosis of primary and/or secondary thyroid lymphoma. We will include reports of adult cases diagnosed with primary thyroid lymphoma or secondary thyroid lymphoma and exclude studies involving patients with primary or secondary tumors of an origin other than thyroid lymphoma. Additionally, studies reporting patients who, alongside primary or secondary thyroid lymphoma, have concomitant diseases associated with high morbidity or mortality, will be excluded. A total of two independent reviewers will participate in the screening, selection, and data extraction process, all of which will be carried out blindly. Conflicts between reviewers will be resolved through discussion or by involving an additional reviewer. Results will be reported using descriptive statistics, and the information will be stored in RedCap (Electronic Research Data Capture).

**Results:**

The literature search was conducted in August 2024, using the following databases: Scopus, MEDLINE, and EMBASE, to identify observational studies. A total of 1404 results were retrieved. The scoping review is expected to be completed by August 2025.

**Conclusions:**

This scoping review will describe the current understanding of the prognostic factors that influence the course of the disease in patients diagnosed with primary and secondary thyroid lymphoma. The review will highlight characteristics that may identify patients at risk of more aggressive disease behavior and could potentially guide therapeutic decisions. Future studies should assess the incidence of these prognostic factors across different age groups and explore their clinical correlations.

## Introduction

Thyroid lymphoma may be classified as primary, when neoplastic cells arise de novo within the thyroid gland, or secondary, when the gland is infiltrated by malignant lymphoid cells originating from other sites [1]. Both entities are rare. Primary thyroid lymphoma (PTL) accounts for approximately 2% of extranodal lymphomas and 1%‐5% of all thyroid malignancies [2]. Diagnosis is established by fine-needle aspiration or open biopsy [3]. Accurate subtype identification is essential, as treatment depends on the histologic variant. Diffuse large B-cell lymphoma (DLBCL) is the most frequent subtype, comprising more than half of reported cases [4], followed by mucosa-associated lymphoid tissue (MALT) lymphoma [5].

In addition, PTL is strongly associated with Hashimoto thyroiditis; several studies suggest that up to 80% of patients with PTL have a background of this autoimmune condition [6]. Prognosis varies according to the lymphoma subtype, with reported 5-year survival rates of up to 45% [7].

In contrast, secondary thyroid lymphoma is an even rarer entity. It may be misdiagnosed as other thyroid pathologies, such as primary lymphoma or intrathyroidal metastases. Definitive diagnosis relies on histopathological evaluation [8]. Takashima et al [9] reported older series in which thyroid involvement among patients with systemic lymphoma ranged from 11% to 27%. Among these, aggressive subtypes such as Burkitt lymphoma often present with rapid progression and poor prognosis if treatment is not initiated promptly [7].

Although the prevalence of both entities is low, early recognition of primary and secondary thyroid lymphomas is essential, as their therapeutic approaches differ substantially from those of other thyroid malignancies. However, published data on these conditions remain limited.

Given this knowledge gap, establishing a comprehensive database that compiles information reported in the literature would be valuable for assessing potential prognostic factors and determining optimal treatment strategies—an endeavor complicated by the rarity of these diseases. The aim of this scoping review is to examine the current literature on primary and secondary thyroid lymphomas and to identify key factors associated with improved patient outcomes

## Methods

The proposed scoping review will be conducted in accordance with the PRISMA-ScR (Preferred Reporting Items for Systematic Reviews and Meta-Analyses Extension for Scoping Reviews) for scoping reviews.

### Review Questions

The research questions for this study are as follows:

- What is the current state of knowledge regarding the clinical course of primary and secondary thyroid lymphoma?
- What is the epidemiology of primary and secondary thyroid lymphoma?
- What are the most frequently observed clinical characteristics in patients with primary and secondary thyroid lymphoma?
- What are the most common histopathological characteristics of primary and secondary thyroid lymphoma?
- What are the most common radiological features of primary and secondary thyroid lymphoma?
- How do these characteristics impact mortality in primary and secondary thyroid lymphoma?
- What clinical factors influence disease progression in primary and secondary thyroid lymphoma?
- What are the existing knowledge gaps and future research directions regarding thyroid lymphoma?

### Participants

This review will consider studies of any design reporting adult patients [18 years or older) with a confirmed diagnosis of primary or secondary thyroid lymphoma (Table 1).

**Table 1.** Inclusion and exclusion criteria for study selection.

Inclusion criteria	Exclusion criteria
Studies reporting adult patients (≥18 years) with a confirmed diagnosis of primary or secondary thyroid lymphoma	Studies describing neoplastic thyroid pathologies other than primary or secondary thyroid lymphoma
Studies including patients with primary or secondary thyroid lymphoma describing frequency, clinical features, and complications	Studies assessing only pediatric populations
Studies providing information on histopathological features	
Studies providing information on treatment strategies	
Studies providing information on factors influencing disease progression and/or mortality in primary or secondary thyroid lymphoma	
Observational and quasi-experimental studies reporting human data	
Studies published in English or Spanish	

### Inclusion Criteria

The following studies will be selected for this review:

- Studies including patients with primary or secondary thyroid lymphoma, describing frequency, clinical features, and complication**s**
- Studies providing information about histopathological features
- Studies providing information about treatment strategies
- Studies providing information about characteristics that condition progression on primary and secondary thyroid lymphoma and mortality

### Exclusion Criteria

The exclusion criteria were as follows:

- Studies describing neoplastic thyroid pathologies other than primary or secondary thyroid lymphoma
- We will exclude studies assessing only children, but allow inclusion of studies describing mixed populations of children and adults

### Concept

The accurate identification of prognostic factors influencing the course of the disease in patients diagnosed with primary or secondary thyroid lymphoma will enable us to determine the characteristics that may indicate a more aggressive disease behavior and even guide therapeutic decision-making. Considering that no studies have systematically compiled this information to date, addressing this gap is essential.

### Context

This review will consider studies on primary or secondary thyroid lymphoma, including population characteristics and histological findings, which may influence treatment selection and survival. Additionally, prognostic factors identified in various databases and search engines will be analyzed.

### Types of Sources

This scoping review will include all sources of scientific evidence, including quasi-experimental and observational studies. No restrictions will be placed on the type of quantitative research included. The review will be conducted following the JMIR methodology for scoping reviews and aligned with the PRISMA-ScR checklist.

### Search Strategy

The search strategy is intended to locate published scientific studies that meet the inclusion criteria. An initial search was performed by the epidemiologist on August 9, 2024, on Scopus (Elsevier), Embase, and Ovid-Medline; this resulted in 1404 article references. A detailed search strategy is provided in Multimedia Appendix 1.

#### Keywords and Index Terms

All key terms were adapted for each included database and information source. The search terms will be developed collaboratively by the entire team of reviewers, but a single reviewer will conduct the search itself. Additionally, the reference lists of all included sources of evidence will be screened for relevant studies. Studies published in Spanish and English will be included (Textbox 1).

Textbox 1. **Search keywords.**

Thyroid lymphoma

Primary thyroid lymphoma

Secondary thyroid lymphoma

### Study or Source of Evidence Selection

A total of 1404 article references were compiled and uploaded to Rayyan, with duplicates removed by the platform and reviewed manually by a researcher. Independent researchers will screen the titles and abstracts, and potentially relevant articles will be retrieved in full, with their citation details imported into the JBI System for the Unified Management, Assessment, and Review of Information.

Full-text articles selected for inclusion will be evaluated against the inclusion and exclusion criteria by the same independent researchers. Reasons for exclusion will be recorded and reported in the scoping review.

Any disagreements between researchers at each stage of the selection process will be resolved through discussion or by consulting a third researcher. The search results will be fully reported in the final scoping review and presented in a PRISMA (Preferred Reporting Items for Systematic Reviews and Meta-Analyses) flow diagram.

### Data Extraction

Data will be extracted from papers included in the scoping review by 2 independent researchers using a spreadsheet to include the following variables [5]:

Title, authors, year of publication, journal, DOI, origin or country of origin (where the source was published or conducted), authors, study type, aims or purpose, population characteristics (age, sex, comorbidities as history of autoimmune thyroiditis); clinical characteristics (Presence of a painless tumor mass in the anterior cervical region, with rapid growth, with or without cervical lymphadenopathy, usually accompanied by symptoms of aerodigestive obstruction); hormonal status (Thyroid profile); imagenologic characteristics; diagnostic method (fine needle aspiration, open biopsy); primary or secondary thyroid lymphoma, if secondary thyroid lymphoma: site of primary lymphoma; classification according to the World Health Organization (WHO) classification of tumors of hematopoietic and lymphoid tissues; immunohistochemical characteristics; presences of other malignancies; surgery (yes or no); treatment (type of chemotherapy, radiotherapy); and overall survival.

The draft spreadsheet will be modified and revised as needed throughout the data extraction process for each included paper. Any modifications will be documented in the final scoping review. Disagreements between researchers will be resolved through discussion or by consulting a third researcher. If necessary, the authors of the included papers will be contacted to request missing or additional data.

### Data Analysis and Presentation

A narrative description of the findings will be discussed in the framework of the review questions. The data will be summarized and presented in tables.

### Reference Searches

Citation tracking will be used to identify key articles relevant to the topic of interest. This process will involve reviewing the reference lists of relevant papers, as well as identifying articles that have cited them. Any duplicate papers that have already been examined will be removed. If a paper meets the inclusion criteria, its reference list will be screened to identify additional relevant manuscripts.

## Results

The search was conducted on August 9, 2024, using Scopus (Elsevier), Embase, and Ovid, yielding a total of 1404 results. A comprehensive review of the manuscripts is expected to be completed by August 2025. All potential manuscripts will be imported into the reference management software Rayyan QCRI 23. This scoping review aims to identify and describe the clinical, histopathological, and molecular prognostic factors associated with survival and disease progression in adult patients with primary and secondary thyroid lymphomas.

**Figure 1.**
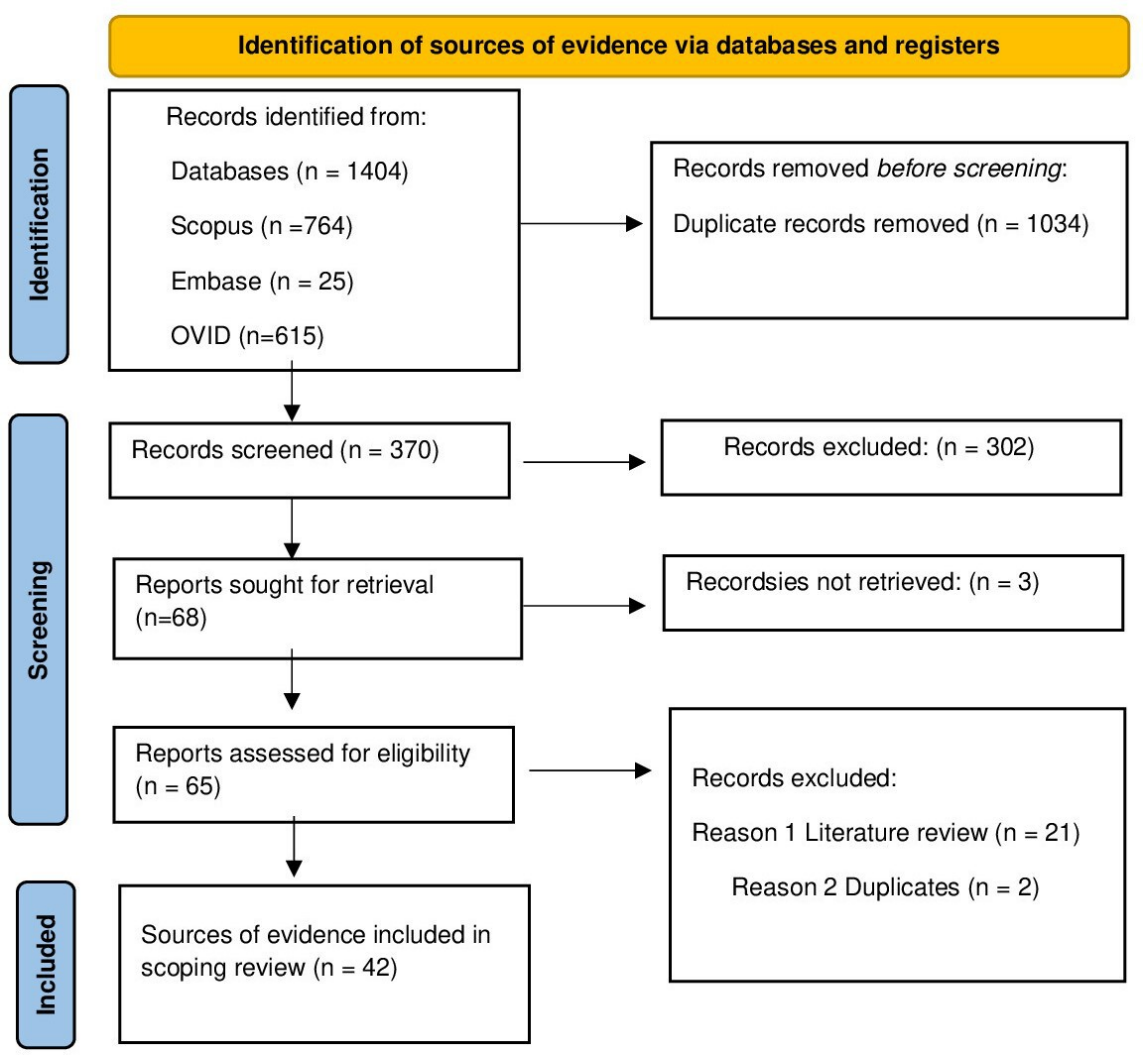
PRISMA (Preferred Reporting Items for Systematic Reviews and Meta-Analyses Extension for Scoping Reviews) flowchart.

A preliminary PRISMA flow diagram illustrating the identification, screening, eligibility, and inclusion process is provided in Figure 1.

## Discussion

Primary and secondary thyroid lymphomas are rare malignant neoplasms that account for only 2%‐5% of all thyroid cancers and approximately 2% of extranodal lymphomas [10,11]. This rarity underscores the need to analyze the various case reports and case series published over time to identify specific characteristics such as histologic subtypes, clinical manifestations, prognosis, and survival, among others [11]. Given their low incidence, the overarching goal is to identify shared features across different patient groups.

With this scoping review, we aim to compile the available observational evidence to determine which clinical factors—such as sex, age, presentation, comorbidities, and disease stage—have been associated with progression and/or survival in patients with thyroid lymphomas [12]. We also aim to identify histopathological characteristics, including histologic subtypes and immunohistochemical markers such as tumor protein p53 (p53) and marker of proliferation Ki-67 (Ki-67), that have been linked to prognosis. This will help confirm that the most frequent histologic subtypes of thyroid lymphoma are DLBCL and MALT, as described in multiple case series [6].

Additionally, we seek to characterize the most relevant and common clinical manifestations—such as dysphagia, hoarseness, and airway obstruction, particularly in rapidly growing masses [13]—to support earlier diagnosis when clinical suspicion arises. However, molecular evidence remains fragmented and heterogeneous, and prognostic factors for secondary thyroid lymphomas are even less consistent and infrequently reported.

Among the main findings, the most frequently evaluated variables across studies include age, the presence of compressive symptoms, a prior diagnosis or documented history of Hashimoto thyroiditis [14], and the different Ann Arbor stages. Certain factors appear to have a more consistent association with survival outcomes[15], including age, tumor size, and the presence of B symptoms [16]. Nonetheless, only a limited number of studies provide comprehensive data on comorbidities at diagnosis [14,17], diagnostic methods [7,17,18], clinical status, and other relevant variables [19].

Regarding the prognostic value of immunomarkers, Ki-67 is perhaps the most extensively studied and most consistently associated with disease aggressiveness. Other markers such as p53, B-cell lymphoma 2 (BCL2), and MYC proto-oncogene (MYC) show variable findings [20], and no adequately standardized protocol exists for their assessment. Molecular factors, including gene rearrangements, are infrequently reported in patients with thyroid lymphoma, making it even more challenging to evaluate somatic mutations or advanced genetic alterations in these tumors [21]. These findings often lack external validation [22], further limiting their incorporation into broader analyses.

This scoping review seeks to highlight the lack of standardization in the characterization of primary and secondary thyroid lymphomas. Examples include the frequent aggregation of primary and secondary cases in previous studies, differences in therapeutic management, and considerable variability in the cut-off values used for Ki-67 [23,24].

Among the strengths of this review, we identify the opportunity to expand the clinical, histologic, and molecular characterization of thyroid lymphomas by including studies with diverse designs and by identifying gaps that had not been clearly documented or systematically addressed in previous literature. Nevertheless, heterogeneity in study designs, sample sizes, and disease stages limits the ability to establish causal relationships. Most of the available evidence is derived from small retrospective series; however, these still contribute valuable insights and facilitate a more structured understanding of the current literature. One of the most significant limitations remains the scarcity of studies focusing specifically on secondary thyroid lymphoma.

These findings underscore the need for prospective multicenter studies that jointly evaluate clinical and molecular factors, standardize immunohistochemical markers and their quantification, and, in the long term, conduct comparative analyses to better establish prognostic differences between primary and secondary thyroid lymphomas.

We hope that disseminating this compiled information and publishing our results in open-access repositories and medical literature will support clinicians and pathologists seeking a deeper prognostic understanding of patients affected by this condition.

This scoping review, therefore, aims to emphasize the importance of clinical and histopathological factors as the predictors with the strongest available evidence in thyroid lymphomas, whereas molecular factors remain fragmented due to their low reporting frequency across studies, making them appear insufficiently explored. Although, as previously mentioned, methodological variability limits the ability to draw solid comparisons, this work constitutes an initial step toward identifying priority areas for standardization and knowledge gaps, providing a clearer path for future research and ultimately improving the prognostic characterization of this rare entity.

## Supplementary material

10.2196/73164 Multimedia Appendix 1 Search strategy thyroid lymphoma.

## Abbreviations

DLBCL: diffuse large B-cell lymphoma
MALT: mucosa-associated lymphoid tissue
PRISMA: Preferred Reporting Items for Systematic Reviews and Meta-Analyses
PRISMA-ScR: Preferred Reporting Items for Systematic Reviews and Meta-Analyses Extension for Scoping Reviews
PTL: primary thyroid lymphoma
