# Supplementary material for: Clinical, Histopathological, and Molecular Prognostic Factors Associated With Survival and Disease Progression in Adult Patients With Primary and Secondary Thyroid Lymphomas: Scoping Review Protocol
Source: JMIR Res Protoc. 2026 Feb 20;15:e73164. doi: 10.2196/73164 (PMC12923092; doi:10.2196/73164)
Supplement: Multimedia Appendix 1 [file resprot-v15-e73164-s001.docx]

| **#** | **Search** | **Results 08/09/ 2024** |
| --- | --- | --- |
| 1 | "Thyroid lymphoma".mp. [mp=title, book title, abstract, original title, name of substance word, subject heading word, floating sub-heading word, keyword heading word, organism supplementary concept word, protocol supplementary concept word, rare disease supplementary concept word, unique identifier, synonyms, population supplementary concept word, anatomy supplementary concept word] | 505 |
| 2 | "Secondary thyroid Lymphoma".mp. [mp=title, book title, abstract, original title, name of substance word, subject heading word, floating sub-heading word, keyword heading word, organism supplementary concept word, protocol supplementary concept word, rare disease supplementary concept word, unique identifier, synonyms, population supplementary concept word, anatomy supplementary concept word] | 5 |
| 3 | "Primary thyroid lymphoma".mp. [mp=title, book title, abstract, original title, name of substance word, subject heading word, floating sub-heading word, keyword heading word, organism supplementary concept word, protocol supplementary concept word, rare disease supplementary concept word, unique identifier, synonyms, population supplementary concept word, anatomy supplementary concept word] | 277 |
| 4 | "lymphoma of thyroid".mp. [mp=title, book title, abstract, original title, name of substance word, subject heading word, floating sub-heading word, keyword heading word, organism supplementary concept word, protocol supplementary concept word, rare disease supplementary concept word, unique identifier, synonyms, population supplementary concept word, anatomy supplementary concept word] | 138 |
| 5 | 1 or 2 or 3 or 4 | 615 |

SCOPUS

|  | "Thyroid lymphoma" OR "Secondary thyroid Lymphoma" OR "Primary thyroid lymphoma" OR "lymphoma of thyroid" | 762 |
| --- | --- | --- |

Embase

|  | 'thyroid lymphoma'/exp OR 'thyroid lymphoma' OR 'secondary thyroid lymphoma' OR 'primary thyroid lymphoma'/exp OR 'primary thyroid lymphoma' OR 'lymphoma of thyroid'/exp OR 'lymphoma of thyroid' | 853 |
| --- | --- | --- |

PubMed

(("primaries"[All Fields] OR "primary"[All Fields]) AND ("thyroid usp"[Supplementary Concept] OR "thyroid usp"[All Fields] OR "thyroid"[All Fields] OR "thyroid gland"[MeSH Terms] OR ("thyroid"[All Fields] AND "gland"[All Fields]) OR "thyroid gland"[All Fields] OR "thyroid usp"[MeSH Terms] OR ("thyroid"[All Fields] AND "usp"[All Fields]) OR "thyroids"[All Fields] OR "thyroid s"[All Fields] OR "thyroidal"[All Fields] OR "thyroideal"[All Fields] OR "thyroidism"[All Fields] OR "thyroiditis"[MeSH Terms] OR "thyroiditis"[All Fields] OR "thyroiditides"[All Fields]) AND ("lymphoma"[MeSH Terms] OR "lymphoma"[All Fields] OR "lymphomas"[All Fields] OR "lymphoma s"[All Fields])) OR (("thyroid usp"[Supplementary Concept] OR "thyroid usp"[All Fields] OR "thyroid"[All Fields] OR "thyroid gland"[MeSH Terms] OR ("thyroid"[All Fields] AND "gland"[All Fields]) OR "thyroid gland"[All Fields] OR "thyroid usp"[MeSH Terms] OR ("thyroid"[All Fields] AND "usp"[All Fields]) OR "thyroids"[All Fields] OR "thyroid s"[All Fields] OR "thyroidal"[All Fields] OR "thyroideal"[All Fields] OR "thyroidism"[All Fields] OR "thyroiditis"[MeSH Terms] OR "thyroiditis"[All Fields] OR "thyroiditides"[All Fields]) AND "lymphoma"[MeSH Terms]) OR (("neoplasm metastasis"[MeSH Terms] OR ("neoplasm"[All Fields] AND "metastasis"[All Fields]) OR "neoplasm metastasis"[All Fields] OR "secondaries"[All Fields] OR "secondary"[MeSH Subheading] OR "secondary"[All Fields]) AND ("thyroid usp"[Supplementary Concept] OR "thyroid usp"[All Fields] OR "thyroid"[All Fields] OR "thyroid gland"[MeSH Terms] OR ("thyroid"[All Fields] AND "gland"[All Fields]) OR "thyroid gland"[All Fields] OR "thyroid usp"[MeSH Terms] OR ("thyroid"[All Fields] AND "usp"[All Fields]) OR "thyroids"[All Fields] OR "thyroid s"[All Fields] OR "thyroidal"[All Fields] OR "thyroideal"[All Fields] OR "thyroidism"[All Fields] OR "thyroiditis"[MeSH Terms] OR "thyroiditis"[All Fields] OR "thyroiditides"[All Fields]) AND ("lymphoma"[MeSH Terms] OR "lymphoma"[All Fields] OR "lymphomas"[All Fields] OR "lymphoma s"[All Fields]))
